# Virus Immune Evasion: New Mechanism and Implications in Disease Outcome

**DOI:** 10.1155/2012/490549

**Published:** 2012-09-24

**Authors:** Rika Draenert, John Frater, Julia G. Prado

**Affiliations:** ^1^Medical Poliklinik, Ludwig-Maximilians University, 80539 Munich, Germany; ^2^Peter Medawar Building for Pathogen Research, Oxford University, Oxford OX1 3SY, UK; ^3^AIDS Research Institute Irsicaixa, Hospital Universitari Germans Trias i Pujol, Badalona, 08916 Barcelona, Spain

The artists of immune evasion in nature are viruses, as ancient as their organic counterparts, these infectious pseudoorganisms have coevolved coupling human and animal evolution during millions of years. Thus, virus replication is based on an efficient recruitment of cell functions through interactions between cellular and viral proteins. As consequence, during their long evolutionary race the immune system has specialized to control pathogens, and virus has specialized to counteract immune responses favour in the battle by their rapid adaptation against environmental changes.

In this special issue, we summarize some of the current knowledge in immune evasion strategies developped by both DNA and RNA viruses. Virus explored and exploits multiple components of the cellular biogenesis and immune signalling. Thus, virus release and infection are enhanced through the convergence of viral and cellular pathways as summarized by T. Wurdinger et al. Alternative mechanisms of chemokine receptors capture (A. G. Jensen et al.), interference of immune signalling cascades and natural killer responses (G. J. Kotwal et al. and C. I. Odom et al.) are some of the strategies acquire for herpesvirus, poxvirus or retrovirus among others (S. N. Schelkunov et al.), to counteract both innate and adaptative host immunity. 

Additionally, we cannot forget that globally virus are the causative agent of multiple diseases leading to worldwide pandemics and associated with multiple human malignancies (S. Chapenko et al.). The aim of this special issue is to put into perspective the need to understand virus immune escape based on a deep knowledge of viral strategies in order to develop new vaccines and therapeutic approaches (B. Reinhart et al.). In this context, the design of new interventions should be truly innovative and effective in order to reduce mortality and mobility rates associated with viral infection in humans.

*Rika Draenert* *John Frater* *Julia G. Prado*

